# The relationships between acetylcholine-induced chest pain, objective measures of coronary vascular function and symptom status

**DOI:** 10.3389/fcvm.2023.1217731

**Published:** 2023-08-31

**Authors:** Steven E. S. Miner, Mary C. McCarthy, Chris I. Ardern, Chris G. R. Perry, Olga Toleva, Lynne E. Nield, Cedric Manlhiot, Warren J. Cantor

**Affiliations:** ^1^ Division of Cardiology, Southlake Regional Health Centre, Newmarket, ON, Canada; ^2^ School of Kinesiology and Health Science, Muscle Health Research Centre, York University, Toronto, ON, Canada; ^3^ Department of Medicine, University of Toronto, Toronto, ON, Canada; ^4^ Department of Cardiology, Emory University, Atlanta, GA, United States; ^5^ The Blalock-Taussig-Thomas Pediatric and Congenital Heart Center, Department of Pediatrics, Johns Hopkins University, Baltimore, MD, United States

**Keywords:** stable angina, fractional flow reserve, microvascular angina, microvascular dysfunction, coronary endothelial dysfunction, vasospastic angina

## Abstract

**Background:**

Acetylcholine-induced chest pain is routinely measured during the assessment of microvascular function.

**Aims:**

The aim was to determine the relationships between acetylcholine-induced chest pain and both symptom burden and objective measures of vascular function.

**Methods:**

In patients with angina but no obstructive coronary artery disease, invasive studies determined the presence or absence of chest pain during both acetylcholine and adenosine infusion. Thermodilution-derived coronary blood flow (CBF) and index of microvascular resistance (IMR) was determined at rest and during both acetylcholine and adenosine infusion. Patients with epicardial spasm (>90%) were excluded; vasoconstriction between 20% and 90% was considered endothelial dysfunction.

**Results:**

Eighty-seven patients met the inclusion criteria. Of these 52 patients (60%) experienced chest pain during acetylcholine while 35 (40%) did not. Those with acetylcholine-induced chest pain demonstrated: (1) Increased CBF at rest (1.6 ± 0.7 vs. 1.2 ± 0.4, *p* = 0.004) (2) Decreased IMR with acetylcholine (acetylcholine-IMR = 29.7 ± 16.3 vs. 40.4 ± 17.1, *p* = 0.004), (3) Equivalent IMR following adenosine (Adenosine-IMR: 21.1 ± 10.7 vs. 21.8 ± 8.2, *p* = 0.76), (4) Increased adenosine-induced chest pain (40/52 = 77% vs. 7/35 = 20%, *p* < 0.0001), (5) Increased chest pain during exercise testing (30/46 = 63% vs. 4/29 = 12%, *p* < 0.00001) with no differences in exercise duration or electrocardiographic changes, and (6) Increased prevalence of epicardial endothelial dysfunction (33/52 = 63% vs. 14/35 = 40%, *p* = 0.03).

**Conclusions:**

After excluding epicardial spasm, acetylcholine-induced chest pain is associated with increased pain during exercise and adenosine infusion, increased coronary blood flow at rest, decreased microvascular resistance in response to acetylcholine and increased prevalence of epicardial endothelial dysfunction. These findings raise questions about the mechanisms underlying acetylcholine-induced chest pain.

## Introduction

Depending on the clinical context and cardiology society, the indications for intracoronary acetylcholine (Ach) infusion range from 1 to 2b for the assessment of patients with suspected vasospastic angina (1–5). The diagnosis of epicardial spasm requires angiographic spasm but also concurrent anginal pain and ischemic ST segment changes. In the absence of epicardial spasm, the mechanisms underlying Ach-induced chest pain and electrocardiographic changes are ill defined. Despite this mechanistic uncertainty, it has been hypothesized that Ach-induced chest pain is due to microvascular spasm. The combination of Ach-induced chest pain and electrocardiographic changes is considered diagnostic of microvascular spasm in multiple guidelines (2, 3, 5). This consensus is challenged by a previous study which provided inconsistent correlations with coronary blood flow (6).

The field of coronary vascular function provides metrics such as coronary flow reserve (CFR) and index of microvascular resistance (IMR), which provide objective measures of microvascular function. These metrics have been extended beyond endothelium independent agents such as adenosine to include endothelium dependent agents including Ach and dobutamine (7–9). Epicardial endothelial function in response to Ach is also routinely measured and has prognostic relevance (10).

The purpose of this study was to determine the relationship between Ach-induced chest pain with objective measures of vascular function and with subjective symptoms of chest pain in a variety of settings. We hypothesized that chest pain would not be correlated with microvascular responses to adenosine (endothelium-independent). We further hypothesized that Ach-induced chest pain would be associated with abnormal responses to Ach (endothelium-dependent hyperemic agent) in the form of elevated IMR, reduced CFR and epicardial endothelial dysfunction. Finally we hypothesized that Ach-induced chest pain would be positively associated with patient subjective symptom burden.

## Methods

### Patient selection

This prospective study included men and women with ischemic-sounding chest pain and non-obstructive coronary arteries (defined by epicardial stenosis <50%), who underwent invasive coronary physiology studies between Oct 2019 and April 2021. Every patient had been referred by cardiologists or other cardiovascular specialists to a subspeciality clinic (Cardiovascular Integrated Physiology Program) for the assessment and management of ongoing symptoms presumed to be of cardiac origin.

### Study protocol

This study was conducted in accordance with the principles of the Declaration of Helsinki, and all patients provided written informed consent prior to enrollmen (SRHC protocol S-021-2021).

#### Clinical assessment

Clinical assessments included past medical history and cardiovascular risk factors. Cardiovascular risk factors were determined by concordance of patient report and the assessment of the referring physicians. Symptom status was assessed using a variety of validated metrics. Physical quality of life was assessed using the World Health Organization Quality of Life-BREF assessment tool (11). Graded exercise stress tests were performed using the Bruce protocol with concurrent 12-lead electrocardiography, for subsequent calculation of the Duke Treadmill Score (DTS) (12). Angina frequency was assessed using question #2 of the short version of the Seattle Angina Questionnaire (13). For both the physical quality of life and the Seattle angina burden, a higher score indicates better clinical status. For the purposes of consistency with the DTS and the Seattle questionnaire, the term “angina” is used as a descriptive term to describe symptoms while remaining agnostic about the underlying mechanisms.

#### Coronary physiology testing

Invasive physiology studies were performed using thermodilution techniques according to previously published methods (7, 8).

Coronary blood flow was quantified by averaging the transit time of three, 3cc aliquot injections of room temperature heparinized saline, to obtain the mean transit time (T_mn_). The inverse of the T_mn_ is strongly correlated with direct measurements of coronary blood flow (14, 15). IMR (IMR = P_d_ x T_mn_) and CFR (CFR = baseline T_mn_/hyperemic T_mn_) were subsequently calculated. Cardiac efficiency was estimated with the ratio of cardiac work to coronary blood flow. Cardiac work was estimated using the rate pressure product. Rate pressure product correlates well with cardiac oxygen consumption and cardiac work (16). The ratio of energy expenditure to myocardial blood flow is an accepted measure of cardiac efficiency (17). Thus cardiac efficiency was calculated as heart rate * systolic blood pressure/(1/T_mn_). Continuous 3-lead electrocardiographic (ECG) monitoring (V1, V2, aVL) was performed during the invasive study. Ischemic ECG changes were defined as transient ST-segment depression or elevation >0.1 mV.

The use of sequential hyperemic agents has previously been validated and published (7, 8). Intravenous adenosine (140 μg/kg/min) and intracoronary Ach (sequential 20 μg test dose followed by 100 μg) were used to assess endothelial independent and dependent microvascular function, respectively. Ach injections were performed a minimum of 3 min after adenosine, to ensure that systemic hemodynamics returned to baseline and patient symptoms dissipated. Ach-IMR was determined immediately following coronary angiography to determine epicardial vasomotion. To clarify the terminology, we refer to the IMR and CFR in response to intravenous adenosine as Ad-IMR and Ad-CFR respectively. Following Ach infusion, we use the terms Ach-IMR and Ach-CFR respectively.

Our lab has demonstrated reliable quality with these techniques. We have shown strong reproducibility of Ad-IMR (*r* > 0.96). The intraclass correlation coefficient for the three measures of transit time during adenosine and Ach were 0.85 and 0.95, respectively (8). Based on these data we have defined an Ach-IMR > 31 as abnormal (8).

All angiograms were reviewed by a single interventional cardiologist (SM). Epicardial spasm was defined as a new >90% stenosis by visual inspection in a major coronary epicardial vessel following intracoronary Ach infusion. Patients with >90% epicardial spasm were excluded from subsequent analysis. Endothelial dysfunction was defined as new vasoconstriction between 20% and 90% following intracoronary Ach infusion (18).

#### Data and statistical analysis

Analyses were performed using IBM SPSS Statistics 23 (Armonk, NY). Clinical variables between these patient groups were compared using an independent samples *t*-test or a Chi-square test, for continuous and categorical variables, respectively. Significance was defined as *p* < 0.05 (CA).

Multivariable logistic regression analysis was performed with mean imputation of missing values. Variables with a *p* value <0.20 on univariate analysis were initially considered with subsequent removal of non-significant factors (CM).

## Results

**Figure 1:**
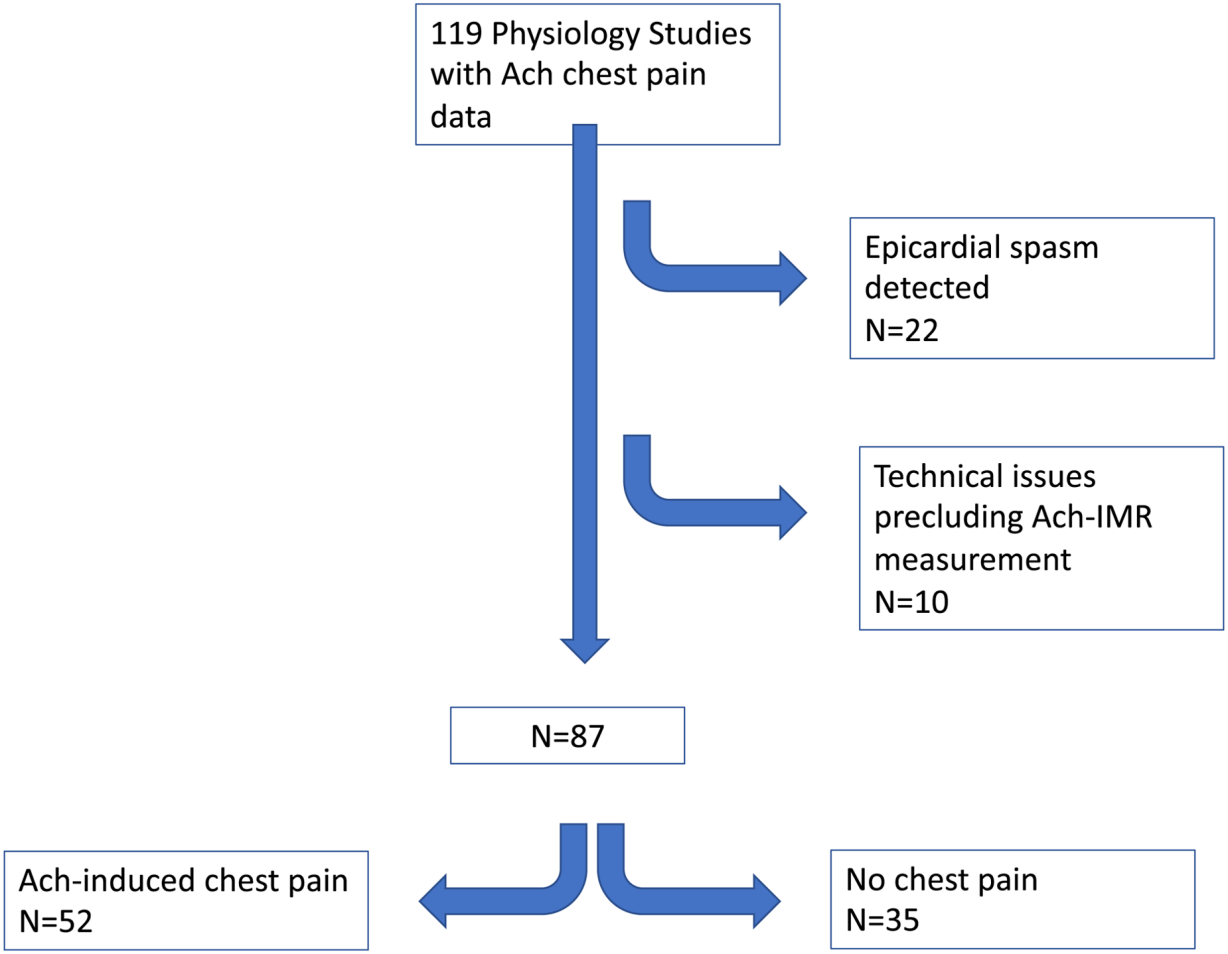
Patient flow. Ach, acetylcholine; IMR, index of microvascular resistance.

Between Oct 2019 and February 2021, 119 patients underwent invasive physiology assessment which included a record of Ach-induced chest pain (see Figure 1). Twenty-two patients were found to have severe epicardial spasm and were excluded. Of the remaining 97 patients, 8 cases were excluded due to technical issues. Of the remaining 89 patients, 2 did not have Ach-IMR recorded at the time of the procedure. Subsequent analysis was performed on the remaining 87 patients.

### Baseline characteristics

The baseline characteristics are presented in Table 1. Patients with Ach-induced chest pain were compared with those who denied chest pain. Patients with Ach-induced chest pain were more likely to have a history of hyperlipidemia and experience chest pain during graded exercise testing. With respect to anginal burden and physical quality of life, patients with Ach-induced chest pain had numerical trends toward worse clinical status that did not reach statistical significance.

**Table 1:** Baseline clinical characteristics of patients with and without acetylcholine-induced chest pain in the absence of epicardial spasm.

Variable	cp +	cp −	*p*
*n* (%)	52 (60%)	35 (40%)	
Age, years	60.7 ± 11.4	59.3 ± 7.9	0.49
Sex, male	17 (33%)	12 (34%)	0.88
Diabetes (51, 35)	10 (20%)	5 (14%)	0.52
Hypertension	28 (55%)	20 (57%)	0.84
Hyperlipidemia	33 (65%)	12 (34%)	0.006
Family History of CAD	12 (23%)	10 (29%)	0.60
Obstructive sleep apnea	9 (18%)	10 (29%)	0.23
Previous MI	13 (25%)	9 (26%)	0.98
Previous CHF	1 (2%)	1 (3%)	0.79
Atrial fibrillation	4 (8%)	2 (6%)	0.70
CRP (mg/L) (42, 25)	3.3 ± 5.1	2.3 ± 1.9	0.23
Beta blockade (%)	42%	45%	0.76
ACE-I or ARB (%)	44%	52%	0.47
Statin (%)	67%	61%	0.63
Ezetimibe (%)	8%	13%	0.51
CCB (%)	46%	35%	0.36
LAN (%)	10%	19%	0.92
Antidepressant (%)	23%	16%	0.46
HbA1c (%) (43, 26)	5.9 ± 0.8	5.9 ± 0.9	0.94
LDL (mmol/L) (45, 26)	2.2 ± 1.0	2.0 ± 0.9	0.51
Uric Acid (mmol/L) (45, 27)	315 ± 96	315 ± 92	0.98
Urine alb/creat (29, 21)	7.8 ± 28.3	1.3 ± 1.2	0.22
GXT ex time (sec) (48, 34)	444 ± 146	434 ± 155	0.77
GXT STD (mm) (46, 33)	0.7 ± 0.7	0.6 ± 0.8	0.72
GXT angina (46, 33)	30 (63%)	4 (12%)	<0.0001
DTS (46, 33)	0.65 ± 5.1	3.89 ± 4.6	0.005
Physical QOL (49, 33)	22.4 ± 5.4	24.0 ± 6.0	0.23
Seattle angina burden percentile (27, 24)	40.0 ± 26.0	55.0 ± 30.8	0.07
LV function			
>60%	26 (50%)	22 (63%)	0.39
40%–59%	25 (48%)	13 (37%)	
30%–39%	1 (2%)	0 (0%)	

MI, myocardial infraction; CHF, congestive heart failure; CRP, C reactive protein; GXT, graded exercise test; STD, ST segment depression during graded exercise testing; DTS, Duke treadmill score; QOL, quality of life; LV, left ventricle; Alb/creat, ratio of urinary albumin to creatinine; HbA1c, hemoglobin A1c; LDL, low density lipoprotein; ACE-I, angiotensin converting enzyme inhibitor; ARB, angiotensin receptor blocker; Statin, HMG CoA reductase inhibitor; CCB, calcium channel blocker; LAN, long acting nitrate.

### Coronary blood flow, cardiac efficiency and microvascular resistance

Patients with Ach-induced chest pain had significantly increased baseline coronary blood flow (1/T_mn_ = 1.6 ± 0.7 vs. 1.2 ± 0.4, *p* = 0.004) (Table 2) reflecting decreased baseline transit times (T_mn_ 0.75 ± 0.33 vs. 0.94 ± 0.33, *p* = 0.009). Baseline cardiac efficiency was lower in patients who subsequently experienced Ach-induced chest pain (7,613 ± 3,250 vs. 9,143 ± 3,610, *p* = 0.04).

**Table 2:** Invasive findings in patients with and without acetylcholine-induced chest pain in the absence of epicardial spasm.

Variable	cp +	cp −	*p*
*N* (%)	52	35	
Baseline CBF (1/s)	1.6 ± 0.7	1.2 ± 0.4	0.004
Baseline cardiac efficiency	7,649 ± 3,219	9,224 ± 4,317	0.03
Endo DysFn (%)	33 (63%)	14 (40%)	0.03
Ad IMR	21.1 ± 10.7	21.8 ± 8.2	0.76
Ad CFR	3.1 ± 1.3	3.7 ± 1.4	0.04
Ad-induced chest pain, *n* (%)	40 (77%)	7 (20%)	<0.0001
Ad-induced ECG changes y/*n* (%)	3/37 (0.08)	3/43 (0.07)	0.86
Ach-induced ECG changes y/*n* (%)	30/22 (58)	14/20 (41)	0.013
Ach IMR	29.7 ± 16.3	40.4 ± 17.1	0.004
Ach CFR	2.5 ± 1.2	2.3 ± 0.8	0.2

Values are mean ± SD or *n* (%). CP +, chest pain in response to acetylcholine infusion; CP −, no chest pain in response to acetylcholine infusion; CBF, coronary blood flow defined as 1/mean transit time; Endo DysFn, endothelial dysfunction defined as new 20%–90% stenosis following intracoronary acetylcholine; Ad-IMR, index of microvascular resistance during intravenous adenosine infusion; Ad-CFR, coronary flow reserve following intravenous adenosine infusion; Ad-induced chest pain, pain during intravenous adenosine infusion; Ach-IMR, microvascular resistance following intracoronary acetylcholine infusion; Ach-CFR, coronary flow reserve following intracoronary acetylcholine infusion; ECG, electrocardiogram; cardiac efficiency, (beats per minute * mmHg)/(baseline CBF = 1/s) = bpm*mmHg*s.

During adenosine infusion, there were no significant differences in Ad-IMR: (21.1 ± 10.7 vs. 21.8 ± 8.2 *p* = 0.76) (Table 2). Coronary flow reserve was significantly lower in patients with Ach-induced chest pain due to the elevated baseline coronary blood flow (Ad-CFR 3.1 ± 1.3 vs. 3.7 ± 1.4, *p* = 0.04). Patients with Ach-induced chest pain were more likely to experience chest pain during intravenous adenosine infusion (40/52 = 77% vs. 7/35 = 20%, *p* < 0.00001). No differences were seen in Ad-induced ECG changes which were infrequent.

During Ach infusion: patients with Ach-induced chest pain had significantly higher coronary blood flow 1/T_mn_ (3.6 ± 1.7 vs. 2.7 ± 1.3, *p* = 0.01), and decreased microvascular resistance Ach-IMR (29.7 ± 16.3 vs. 40.4 ± 17.1, *p* = 0.004). Ach-induced chest pain was associated with a higher rate of Ach-induced ECG changes (58% vs. 41%, *p* = 0.013).

### Endothelial dysfunction and cardiac efficiency

Endothelial dysfunction was more prevalent in patients with Ach-induced chest pain (63% vs. 40%, *p* = 0.03, see Table 2). Patients with endothelial dysfunction had significantly lower Ach-IMR (29.5 ± 16.2 vs. 39.3 ± 17.4 *p* = 0.008) and significantly higher Ach-CFR (2.7 ± 1.2 vs. 2.2 ± 0.8, *p* = 0.04). There were no differences in the microvascular response to adenosine.

Patients with endothelial dysfunction demonstrated a trend toward increased baseline coronary blood flow (1.57 ± 0.7 vs. 1.33 ± 0.57, *p* = 0.08), and significantly reduced cardiac efficiency (7,568 ± 2,926 vs. 9,124 ± 3,767, *p* = 0.03).

**Figure 2:**
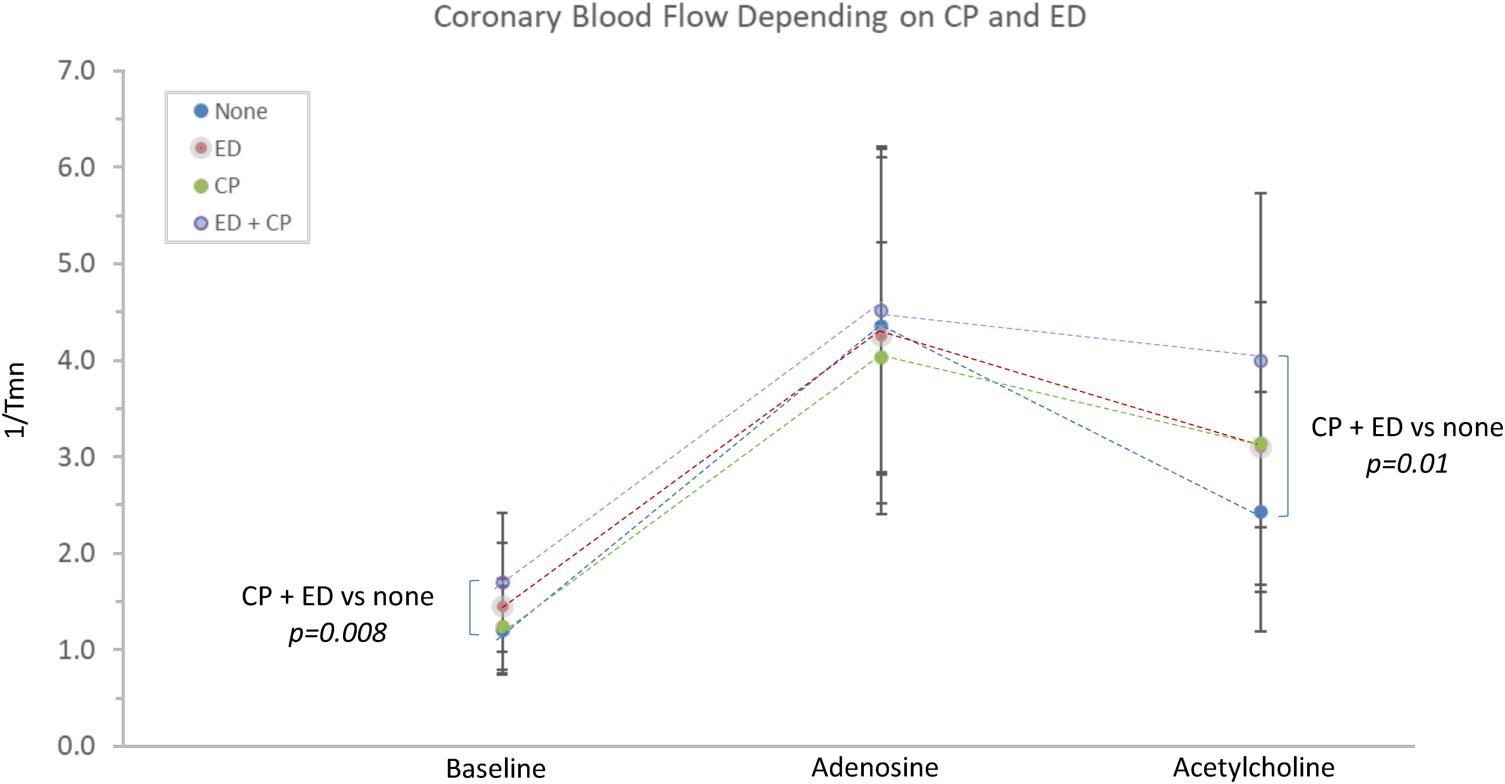
The relationship between Ach-induced chest pain, endothelial dysfunction and coronary blood flow. CP, acetylcholine-induced chest pain; ED, epicardial endothelial dysfunction; Tmn, mean transit time.

**Figure 3:**
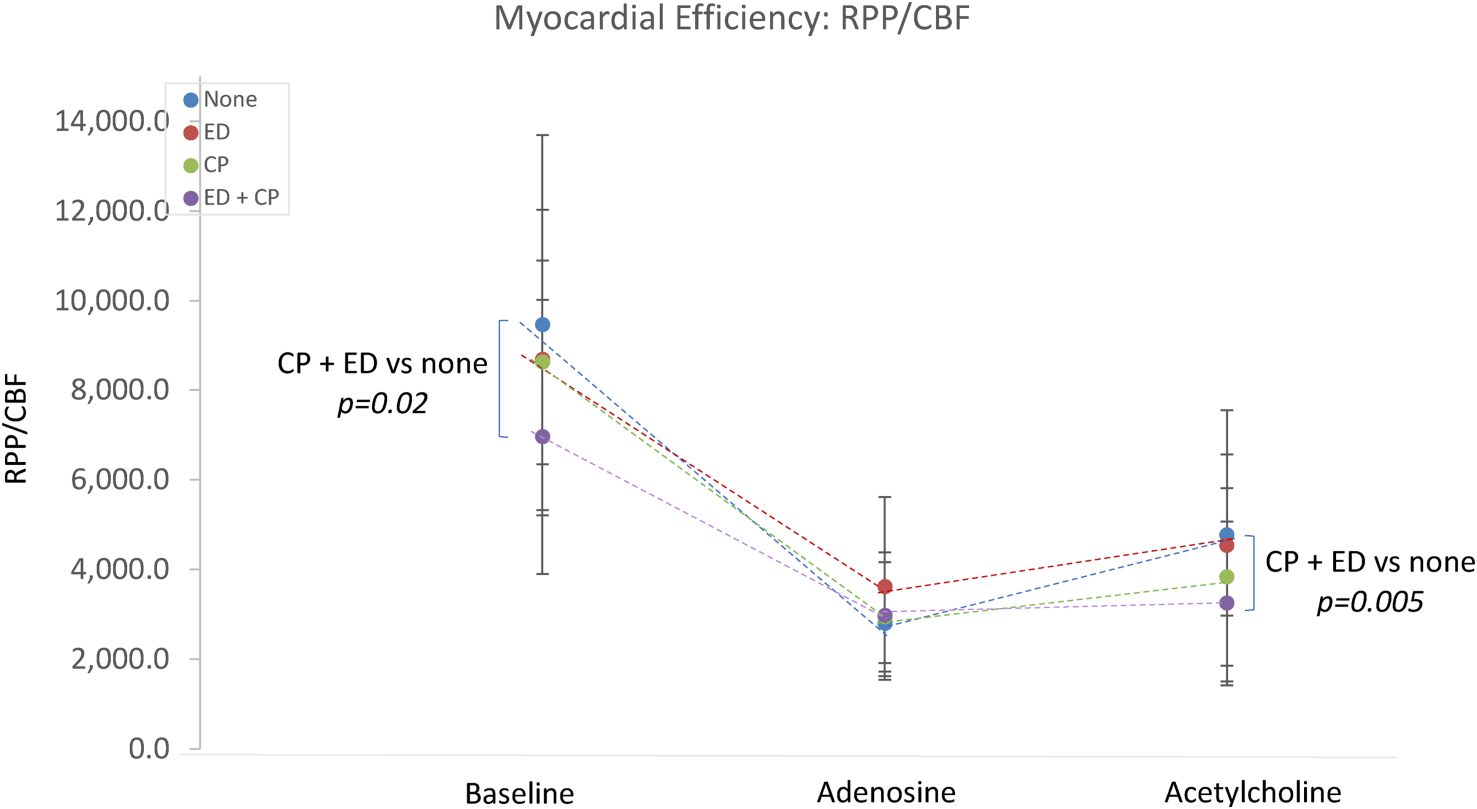
The relationship between Ach-induced chest pain, endothelial dysfunction and cardiac efficiency. CP, acetylcholine-induced chest pain; ED, epicardial endothelial dysfunction; RPP, rate pressure product; CBF, coronary blood flow.

The interaction between epicardial endothelial dysfunction and Ach-induced chest pain was explored. Patients with both (*n* = 33) or neither (*n* = 21) were compared. There were no significant differences in the microvascular response to adenosine. Those with both chest pain and endothelial dysfunction demonstrated significantly lower Ach-IMR (26.7 ± 14.8 vs. 43.4 ± 16.3, *p* = 0.0003) and higher Ach-CFR (2.7 ± 1.3 vs. 2 ± 0.5, *p* = 0.04). The concurrent endothelial dysfunction and Ach-induced chest pain identified patients with the highest baseline coronary blood flow and the highest blood flow in response to intracoronary Ach (Figure 2). This same group also demonstrated the lowest cardiac efficiency at baseline; 7,060 ± 3,086 vs. 9,526 ± 4,148 *p* = 0.02 (Figure 3).

### Multivariable analysis

Multivariable logistic regression analysis is presented in Table 3. Significant predictors of Ach-induced chest pain included Adenosine-induced chest pain, chest pain during exercise testing, lower Ach-IMR and a history of hyperlipidemia.

**Table 3:** Multivariable analysis of Ach-induced chest pain: analysis of maximum likelihood estimates.

Parameter	DF	Estimate	Standard error	Wald chi-square	Pr > ChiSq
Intercept	1	−0.0589	0.8222	0.0051	0.9429
ad_cp_	1	2.6782	0.7840	11.6682	0.0006
ach_imr	1	−0.0696	0.0238	8.5840	0.0034
hyperlipidemia_	1	1.8426	0.7250	6.4601	0.0110
any_gxt_angina	1	1.9039	0.7975	5.6999	0.0170

Ad cp, adenosine-induced chest pain; Ach imr, index of microvascular resistance during acetylcholine induced hyperemia; GXT, graded exercise test.

## Discussion

These data add to the literature by revealing that in the absence of epicardial spasm, Ach-induced chest pain was associated with increased basal coronary blood flow, decreased basal coronary microvascular resistance, decreased cardiac efficiency, and decreased Ach-IMR. It was associated with increased prevalence of adenosine-induced chest pain, increased prevalence of exercise-induced chest pain, and increased prevalence of epicardial endothelial dysfunction. These finding have implications for the mechanistic interpretation on invasive coronary physiology studies.

### Clinical relevance

Approximately 30% of patients with chest pain syndromes have non-obstructive coronary angiograms. Crea and Lanza (19) have noted that this syndrome has multiple potential mechanisms, and there is an opportunity cost to assuming the wrong mechanism in individual patients. Ach-induced chest pain is currently an important part of the diagnostic guidelines. The combination with electrocardiographic changes is assumed to be the result of acute Ach-induced microvascular spasm and to represent an “inappropriate susceptibility to microvascular constriction” (20). The current data does not support this hypothesis. The significantly decreased Ach-IMR is not consistent with widespread microvascular spasm though it does not rule out maldistribution and subendocardial steal due to altered autoregulation (21).

This observation is clinically relevant because vasodilators are routinely recommended for the management of coronary microvascular spasm (18). In the absence of objective measures of increased microvascular resistance (acute or chronic), it is not clear that this strategy has a sound mechanistic underpinning. This may help to explain the failure of diltiazem to improve symptoms in patients with angina but no obstructed coronary arteries (22). Further, the assumption that Ach-induced chest pain represents microvascular spasm distracts from other potential mechanisms that require ongoing investigation.

### Ach-induced chest pain and myocardial ischemia

While Ach-induced chest pain was associated increased overall coronary blood flow, this does not eliminate the possibility of ischemia due to regional perfusion heterogeneity (23). On univariate analysis, Ach-induced chest pain was associated with slightly higher incidence of Ach-induced ECG changes. However, this occurred only 17% more frequently in those with versus without chest pain. In a *post-hoc* analysis, concurrent chest pain and ECG changes were not associated with decreased CBF or increased IMR (data not shown). Independent of chest pain, Ach-induced ECG changes were not associated with significant differences in baseline coronary blood flow, cardiac efficiency, Ad-IMR, Ad-CFR, Ach-IMR or Ach-CFR (data not shown). In multivariable analysis, Ach-induced chest pain was not associated with Ach-induced ECG changes. Ach augments ECG changes via vagal influence in patient with Brugada syndrome (24, 25), and in a canine model induces a variety of ST segment changes (26). Indeed, chest pain and ECG changes are relatively common during coronary angiography without any clear mechanistic explanation (27). Thus ECG changes alone should not be considered diagnostic of ischemia. Transcardiac lactate production might be helpful, but it is important to note that Ach may induce lactate production via mechanisms that are independent of ischemia (28–31). This may explain why a previous subset of patients with both Ach-induced chest pain and ECG changes had transcardiac lactate production despite increased coronary blood flow (6). These data mirror our own. While it is likely that some Ach-induced chest pain is secondary to true myocardial ischemia, the current data suggest that Ach-induced chest pain, with or without ECG changes, may not be sufficiently specific to be used as a criterion for this diagnosis.

### Chest pain and nociceptive disorders

Altered pain sensation is well-documented in patients with chest pain and normal coronary arteries (32). Chronic pain may be associated with abnormal nicotinic Ach receptor activity (33). Sixty percent of our patients complained of chest pain during Ach infusion. Seventy-five percent of these patients also complained of chest pain during adenosine infusion despite normal microvascular responses. They were also more likely to have exercise-induced chest pain without other signs of ischemia. Previous studies have suggested that adenosine induces chest pain “by mechanisms other than myocardial ischemia” (34). In multivariable analysis, adenosine-induced chest pain was the strongest predictor of Ach-induced chest pain. These findings raise the hypothesis that Ach-induced chest pain is a marker for abnormal nociception. Future studies could examine the correlation between Ach- and adenosine-induced chest pain with peripheral pain thresholds (35).

### Chest pain and endothelial dysfunction

Ach-induced chest pain was associated with an increased prevalence of endothelial dysfunction, which in turn was associated with both increased baseline coronary blood flow, and reduced Ach-IMR.

These findings are initially counterintuitive but may be partially explained by the differential effects of Ach on large versus smaller coronary vessels. L-N monomethylargnine (L-NMMA) abolishes the vasodilatation of the epicardial arteries (36) and larger arterioles (37) but only partially inhibits the vasodilatation of the smaller microvasculature. At least 60% of the microvascular vasodilatory effect of Ach is via mechanisms other than nitric oxide (38). Thus, while epicardial vasodilatation (and endothelial dysfunction) is essentially completely dependent upon Ach-induced nitric oxide production, this effect plays a secondary role in the microvasculature. Our intuitively paradoxical findings mirror a previous study in which 48% of hypertensive patients had constriction of the epicardial conduit arteries but vasodilatation of the microvasculature in response to intracoronary Ach (39).

Endothelial dysfunction has also been associated with chronic pain syndromes. Deep somatic sympathetic afferents contribute to pain syndromes, raising the possibility of a direct effect of Ach on nociception in the heart (40). The association between endothelial dysfunction and Ach-induced chest pain may reflect the complex mechanisms underlying chronic pain syndromes.

### Cardiac efficiency

We have previously reported that patients with abnormal CFR but normal IMR have decreased cardiac efficiency (9). Our current data identifies both Ach-induced chest pain and endothelial dysfunction as synergist markers of a similar endotype. Looking specifically at patients with or without chest pain the underlying mechanisms are unclear but it is worth noting that the prevalence of hyperlipidemia was 66% in patients with both chest pain and endothelial dysfunction compared with 29% with neither (*p* = 0.008). We did not measure free fatty acid concentrations, but increased levels are associated with decreased metabolic efficiency and increased coronary blood flow (41) in addition to endothelial dysfunction and the activation of specific pain receptors (42). The potential role of free fatty acids, endothelial dysfunction and myocardial metabolic efficiency should be considered in future studies.

### Clinical relevance of Ach-induced chest pain

These data should not be interpreted as a repudiation of Ach-induced chest pain as a relevant clinical variable. The correlation with decreased cardiac efficiency raises the possibility of impaired myocardial energetics (9). Ach-induced chest pain was associated with epicardial endothelial dysfunction which itself is associated with adverse clinical outcomes (10). The potential association with chronic pain opens new avenues for research (33). These findings suggest that Ach-induced chest pain is not benign and argue for further investigations into the underlying mechanisms. As stated previously, the current data suggest an association with increased coronary blood flow but do not exclude the possibility of regional hypoperfusion. Specific transcardiac markers of ischemia may be required to differentiate these competing mechanisms.

### Limitations

The current study includes only patients with a chest pain syndrome and no obstructive coronary arteries. Thus, the measures of microvascular resistance in the “control” group may not reflect the population at large.

The utilization of thermodilution techniques during Ach provocation for measurement of IMR and CFR requires further validation. The microvascular-specific metric of Ach-IMR is associated with relevant risk factors and non-invasive measures of vascular dysfunction (7, 8), but it's prognostic significance has not yet been demonstrated.

Epicardial vasomotion was determined by visual inspection. Quantitative coronary angiography would add precision to this measurement. This study did not include free fatty acid measurements. Thus, any commentary on free fatty acids are purely speculative.

## Conclusion

The study confirms the independence of the epicardial and microvascular responses to intracoronary Ach. Ach-induced chest pain is associated with increased chest pain in response to both exercise and intravenous adenosine. It is also associated with increased baseline coronary blood flow, decreased cardiac efficiency, increased endothelial dysfunction and reduced microvascular resistance in response to Ach. There appears to be a synergistic relationship between Ach-induced chest pain and epicardial endothelial dysfunction. These findings raise important mechanistic and therapeutic implications.

## Data Availability

The raw data supporting the conclusions of this article will be made available by the authors, without undue reservation.
